# Concerning Stiffness of the Spine

**Published:** 1907-04-13

**Authors:** William Bennett

**Affiliations:** K.C.V.O.


### Hospital Clinics.

### CONCERNING STIFFNESS OF THE SPINE.

By SIR WILLIAM BENNETT, K.C.V.O.

A Post-Graduate Lecture delivered at the London School of Clinical Medicine (Seamen's Hospital).

Gentlemen,—It is, I presume, hardly needful to say that symptoms are not in proportion to the gravity of the disease by which they are caused, since it is common knowledge that grave disease may exist without noticeable symptoms, and that distressing symptoms may be associated with comparatively trivial complaints. It would be difficult to find a better illustration of this fact than that provided by certain cases of rigidity of the spinal column, a condition which, on the one hand, may be a symptom of the greatest moment, and on the other may be of no real importance at all provided that an accurate diagnosis of its causation be arrived at.

The cases included in the following table, which represents only those examples which have come under my personal observation, and is not intended to be a complete list, will show how large a field the question of rigidity of the spine covers, and how many factors are concerned in its production, more I fancy than are ordinarily realised:—

Table of Causes of Stiffness of the Spine Without Visible Deformity, and Exclusive of Acute Conditions.

| | | |
|---|---|---|
| Persistent | | Tuberculous Disease |
| | | Osteo-arthritis |
| | | *Spontaneous Ankylosis (Sepsis?)* |
| | | Effects of Empyema |
| | | Malignant Disease |
| | | Hydatid of Vertebræ |
| | | Aneurysm |
| | | *Old Fracture* |
| | | *Traumatic Myositis Ossificans* |
| | | *Congenital peculiarity* |
| | | Senile rigidity |
| Intermittent or Relapsing | | Neurosis. N.B.—*Neuro-mimesis* |
| | | Chronic Rheumatism, Muscular and Fibrous |
| | Reflex | Worms |
| | | *Movable Kidney* |
| | | *Renal Calculus* |
| | | *Stone in Ureter* |
| | | *Hip Disease* |
| Temporary or Transient | | Early Tuberculosis |
| | | Gumma |
| | | Sprains and Wrenches |
| | | *Rickets* |

Note.—The lines in italics refer to the less commonly recognised causes of stiffness of the spine and are those towards which the remarks in the lecture are mainly directed.

The whole subject is, as can readily be seen, far too large to be dealt with in a single lecture, or indeed in several lectures of this kind; all, therefore, that I propose now to do is to call attention to certain broad features in some of the varieties of spinal stiffness, with a view especially to combat an impression which seems to be far too prevalent, that anything like pronounced rigidity of the spine, in the absence of congenital and acquired deformity, is a strong indication of tuberculous disease—an impression which I have reason to know too often leads to faulty treatment.

### Distribution and Character of Stiffness.

*Distribution of the Rigidity.*—This may involve the whole length of the spine or may be confined to limited areas, *e.g.*, the lumbar, dorsal, or cervical regions separately; in rare cases two distinct areas, *e.g.*, the cervical and dorso-lumbar, may be involved, the intervening parts of the vertebral column retaining their suppleness.

*The Characteristics of the Stiffness.*—(1) The stiffness may be absolute, that is to say, the spine or the portion of it involved may be rigid like a bar of iron, even when the patient is anæsthetised. (2) The stiffness may be "sub-absolute," that is to say, complete in ordinary circumstances, but disappearing under the influence of an anæsthetic, the rigidity recurring upon the recovery from the anæsthesia. (3) The stiffness, although at first apparently absolute, becomes, upon proper examination, modified, with the result that the spine begins to "give" in a succession of jerks in consequence of the stammering of the muscles—a condition which I have ventured to call the "stammering spine." (4) The stiffness may be, as indicated in the table, persistent, intermittent or relapsing, and temporary or transient.

### Persistent Stiffness.

Absolute Rigidity.—This may be divided into two varieties, that which is unaffected by the administration of an anæsthetic, and that which disappears under anæsthesia. These cases may again be considered under two heads—those occurring during adolescence and under the age of twenty-five, and those occurring later in life. In the former, if the rigidity persists under anæsthesia, the cause is probably either tuberculous disease or congenital peculiarity. In tuberculosis other symptoms are generally sufficient to settle the question, but it is a mistake to suppose that the rigidity of tuberculosis is always associated with pain or tenderness, as sometimes both symptoms are wanting.

It is somewhat surprising that congenital stiffness of the spine, affecting almost invariably the lumbar or dorso-lumbar region, is so little recognised, as it is not altogether rare; the importance of a proper estimate of it is obvious. The following example is sufficient to show this: Whilst a girl eight or nine years old was taking her bath her mother thought that she moved as if her back was stiff, and therefore sought advice of a medical man who found complete immobility of the vertebræ in the lumbar and lower dorsal region without any deformity. The child seemed perfectly well, had been playing hockey and other games, and showed no signs of any defect. At the same time the rigidity was naturally a cause of anxiety, and the patient was referred to me. Upon examination the lumbar spine was absolutely rigid, there was neither pain, tenderness, nor deformity; in walking there was a slightly peculiar gait, unnoticeable under ordinary circumstances. Having seen cases before of the same kind, notably one recorded in the "Clinical Journal" of November 11, 1903, I at once suspected the cause of the condition, and, upon examining other members of the family, found the same peculiarity in two of them. The importance of recognising such a condition is clear, as failing such a recognition the temptation to err on the side of safety by treating a case of the kind as one of tuberculosis would be very strong. In a case, therefore, of stiffness of the spine without pain or deformity in a young subject, I never fail to inquire carefully into the condition of the spines of other members of the family.

In the absolute stiffness of old people, osteo-arthritis or spontaneous ossification, may, in the absence of other distinct indications, be assumed to be the cause, due allowance, of course, being made for the rigidity being possibly due to cured conditions (*e.g.*, tubercle) in early life.

### A Characteristic Case.

In the intermediate period between adolescence and old age a variety of causes such as those enumerated in the table, may account for the condition, and the clinical symptoms are generally sufficient to prevent any mistake in the diagnosis. The revelation of the *x*-rays in injury to the cervical spine makes it clear that fracture of the spine, principally affecting the laminæ, but sometimes involving the bodies, ending in recovery, is more common than has hitherto been supposed, and that such cases are followed by stiffness, which varies, of course, according to the severity and distribution of the injury. I have at the present time a case under observation which illustrates this condition well; the patient is a man approaching 35 years of age, and the cause was a hunting accident. In persistent stiffness following injury traumatic myositis ossificans must not be overlooked as a cause, as although the disease does not, of course, affect the spine itself, the conversion of adjacent muscle into a calcified mass may cause a rigidity as firm as if the spine itself were involved. The following is a characteristic case:—A man 35 years old slipped downstairs in running to catch a train. Although a good deal shaken, he went about his business at once in the ordinary way, but was unable to resume it on the following day in consequence of the pain in his back "across the kidneys"—he seems to have been laid up for some months, "on and off," but finally got sufficiently well to do what was absolutely necessary in the way of getting about—his back, however, always remained stiff, bending being difficult and in wet weather painful. For this stiffness he consulted me some three years after the accilent, not having previously seen a medical man for about two years. On examination of the spine was absolutely stiff over the lumbar region, the dorsal portion being supple. There was no pain, tenderness, or any visible abnormal condition, but on the right side of the lumbar spine, corresponding in outline and situation to the quadratus lumborum muscle, over which the erector spinæ seemed harder than usual, was a mass of stony consistence, quite insensitive and absolutely fixed; the lumbar spine and the mass moved as a solid whole. The *x*-rays showed an opaque area corresponding to the mass mentioned, the nature of which admitted of no doubt. Nothing, of course, could be done beyond telling the patient to make the best of the situation and to get as much compensatory movement in the parts by means of exercise as was feasible. I afterwards heard that a diagnosis of malignant growth had been made, but this was obviously the result of either an imperfect examination or because the practitioner concerned had previously had no experience of such cases. These masses, resulting from traumatic myositis ossifican are, in some respects, it is true, suggestive of a growth; indeed, operations have been proposed in some cases in consequence, but to any person familiar with the condition the mistake is not likely to arise.

### A Clinical Sign.

2. *The Stammering Spine.*—In the condition ferred to in the foregoing remarks the rigidity is so complete that there is little probability of any mistake occurring. There is, however, a form of persistent rigidity which, although only apparent, is at first sight so absolute that unless an examination of the right kind be made, errors in diagnosis are more than probable. This form of rigidity is due to muscular contraction or spasm, and is most frequently met with in muscular rheumatism, hysteria, neuro-mimensis, and as a consequence of reflex irritation (*vide* table), and so comsometimes is the fixation of the vertebræ that a condition of absolute rigidity is produced. Bearing in mind the essential factor—muscular contraction—in these cases, it is obvious that any method of examination of a rigid spine should be made with a view to eliminate the question of deception from this condition.

Excepting cases of inflammation, congenital abnormality, or central nervous disease, it will be found that when stiffness of a joint, vertebral or otherwise, is due to muscular spasm, relaxation of the muscles concerned for a longer or shorter period is merely a question of putting strain upon them for a sufficiently long time. The relaxation may be only momentary, but it will come, and however short the term of relaxation may be, it is sufficient to provide the key to the situation.

The administration of an anæsthetic would, of course, at once settle the question of the relation of muscular spasm to the rigidity; but it should be borne in mind that the anæsthetic, although it may set at rest the question of rigidity, often veils, for the time being, other symptoms of importance. Moreover, the administration of an anæsthetic unnecessarily is not to be commended. In the examination to determine the points under discussion, all that is necessary is that the patient, stripped to the hips, standing with the back to the observer, should bend forward in the usual way, the lower limbs being kept extended at the knee; the hand of the observer at the same time resting upon the rigid portion of the spine. The bending forward of the trunk having been carried out as far as possible, the patient should not immediately begin to resume the upright position, as is usually done in the examination of spinal cases, but should maintain the bent position for a considerable period, at all events for two or three minutes. It will then be found that if the rigidity is due to muscular action, a slight quivering of the muscles lying under the hand of the investigator will occur; this quiver will, if the bent position be still maintained, be replaced by a series of jerks (stammering), until in some cases the flexibility of the vertebral column can be freely established. In infants and the very young this method is obviously unpracticable, but in such the same end is attained by resorting to the only delicate test of spinal rigidity available, namely, by placing the small patient flat on the back and lifting it off the couch or bed by one hand placed beneath the spine; when held up in this way for a minute or so, if the rigidity is due to muscular causes, the result mentioned will follow. So far as I know, this method of testing the rigidity or flexibility of the spine of infants or very young children is the only truly reliable one. The method of placing the child in the prone position and moving its lower limbs and pelvis from side to side is far less sensitive. I know of no more important point in the diagnosis of rigidity of the spine than that afforded by the "stammering spine," and it is a point which may altogether escape notice if the patient, whilst being examined, is allowed, after bending in the orthodox manner, to resume the erect position immediately.

### Some Illustrative Cases.

3. (*a*) A boy, just passing from the adolescent period, of a markedly neurotic type, and the offspring of somewhat "highly strung" parents, was reported to have had a slight accident in the football field, followed by some pain and stiffness in the lumbar region. He apparently recovered, but as he walked rather stiffly and became easily tired, he was seen by a medical man, who, finding rigidity of the spine, came to the conclusion that the lad was the subject of tuberculous disease, and advised complete rest on the back for a period of twelve months, to be followed by the use of a spinal jacket. As this rather drastic suggestion came upon the parents as a complete surprise, they were reluctant at first to resort to the treatment without the support of a further surgical opinion. The physician concerned in the case consequently referred the boy to me. Upon examination, he was found to be an elongated "weedy" looking subject, obviously still suffering from the nervousness of adolescence; discomfort in the lower part of the back was complained of, and he tired very quickly after extertion; he preferred to rest or loll on a sofa, etc., rather than to exert himself, on the grounds of the discomfort, sometimes amounting to pain, which followed any but the slightest efforts at movement. The whole of the lumbar and dorsal spine was stiff; at first sight absolutely so, but upon applying the test I have described a distinct quivering in the erector spinæ soon commenced, and the typical stammering movements followed. Further careful examination having failed to elicit any other evidence whatever of tubercle, I advised massage and rational exercise under proper supervision; these were subsequently carried out, with the result that in a couple of months the boy was sound and strong. He went at once to a public school, and never ailed anything afterwards, although it is some years since the treatment was carried out. It is clear that in this case a knowledge of the stammering symptoms in rigidity of the spine prevented the adoption of a treatment of long-continued confinement and rest, which from every point of view would have been bad.

(*b*) A middle-aged man, who came of a tuberculous stock, complained of a very intractable kind of lumbago, which persisted so long as to become a serious obstacle to business. Upon examination the lumbar spine was apparently quite stiff, and remained so during the ordinary test of bending with immediate return to the erect position. The erectors spinæ were very hard and rigid. Under the stammering test, however, the usual quiver in the muscles, followed by subsequent jerkings, was felt. The question of tuberculous disease having been thus eliminated the patient was sent to a continental watering-place and returned in good health. The occurrence of this symptom of stammering may be held to be absolute proof of the absence of organic disease of the vertebral column.

### The Stammering Jerk.

It is interesting to note, although I do not know that the fact is of any special clinical value, that in the stammering spine the jerks occur in two forms, in the one the first jerk, which is brief, is followed by a succession of increasingly long relaxations in the muscles until sometimes a condition of nearly natural suppleness is arrived at. The process may, in fact, be diagramatically expressed by the following markings:

— —— ——— —————

In the other form the first muscular relaxation is long, those succeeding becoming shorter and shorter until, in some cases, a condition of apparently complete rigidity may be resumed, the diagrammatic expression being thus:

————— ——— —— —

The former type is, so far as I know, limited to cases of "hysteria" and neuro-mimesis, the latter is mainly confined to cases of rheumatism and recent sprains and wrenches.

(*To be continued.*)

Refuse Destructors.—A refuse destructor is an absolute necessity in every well regulated institution where a large amount of dangerous or putrescible waste is produced. Nowadays there are many types of the forced draught combustion destructors on the market. Messrs. Meldrum Brothers, Ltd., of Timperley, Manchester (London offices, 66 Victoria Street, S.W.), make a speciality of small high-temperature destructors, and their new catalogue, which is profusely illustrated, gives details of several economical models. We have seen their apparatus at work in the various institutions under the control of the Metropolitan Asylums Board, and can bear witness to their efficiency. The catalogue, which is more than a mere enumeration of prices and measurements, gives interesting details of the various models which the firm supplies and of their working.

